# Caregiver Services: Needs, Benefits, and Best Practices

**DOI:** 10.1093/geroni/igab046.1187

**Published:** 2021-12-17

**Authors:** Susan Reinhard

**Affiliations:** AARP Public Policy Institute, Washington, District of Columbia, United States

## Abstract

Just under 1 in 5 Americans (19.2%) are caregivers for adults with chronic illnesses. Caregiving is the great equalizer as caregiving remains an activity that occurs among all generations, racial/ethnic groups, income or educational levels, family types, gender identities, and sexual orientations. This presentation will provide a snapshot of the current status of caregiving in the United States. It will explore why caregiver services are needed and will highlight the impacts many caregivers face as a result of their stepping up to help family and friends. In addition, this presentation will discuss what is considered best practice in caregiver services and how the public and private sectors can work together to develop solutions to support family caregivers and those under their care.

